# Soft document clustering using a novel graph covering approach

**DOI:** 10.1186/s13040-018-0172-x

**Published:** 2018-06-14

**Authors:** Jens Dörpinghaus, Sebastian Schaaf, Marc Jacobs

**Affiliations:** Fraunhofer Institute for Algorithms and Scientific Computing, Sankt Augustin, Schloss Birlinghoven, Sankt Augustin, Germany

**Keywords:** Document clustering, Information retrieval, Graph theory

## Abstract

**Background:**

In text mining, document clustering describes the efforts to assign unstructured documents to clusters, which in turn usually refer to topics. Clustering is widely used in science for data retrieval and organisation.

**Results:**

In this paper we present and discuss a novel graph-theoretical approach for document clustering and its application on a real-world data set. We will show that the well-known graph partition to stable sets or cliques can be generalized to pseudostable sets or pseudocliques. This allows to perform a soft clustering as well as a hard clustering. The software is freely available on GitHub.

**Conclusions:**

The presented integer linear programming as well as the greedy approach for this $\mathcal {NP}$-complete problem lead to valuable results on random instances and some real-world data for different similarity measures. We could show that PS-Document Clustering is a remarkable approach to document clustering and opens the complete toolbox of graph theory to this field.

**Electronic supplementary material:**

The online version of this article (10.1186/s13040-018-0172-x) contains supplementary material, which is available to authorized users.

## Background

Soft Document Clustering using a graph partition in multiple pseudostable sets has been introduced in [1]. We would like to extend this approach by making some fundamental theoretical additions, discuss the correct calculation of the bounds *ε* and *ι* and discuss some output data. In addition, we will present a divide and conquer approach to parallelise the computation and reduce the runtime on big instances.

*Document Clustering* (also known as *Text Clustering*) is a specific application of textmining and a sub-problem of cluster analyses. If the categories for sorting are given, it is called *Text Classification Problem*. The approach discussed in this paper can also be applied to other clustering subjects, but the purpose of text clustering is the most common. The application of Document Clustering is a wide and open field and in terms of complexity it is still under heavy research, see [2, 65ff] and [3, 47].

Document Clustering is usually not perceived as a graph problem. We will discuss, how we can generalize this problem so that it is a graph-theoretical problem. Thus, following [4] we would like to split the process into two steps. At first we need to define a similarity measure appropriate to the data domain. Then the technical clustering process can be done using a graph-theoretical approach. Jain et al. also suggested a last step called “assessment of output”. We will show that this can also be solved using graph theory and building the graph visualization proposed in this paper. The Cluster Hypotheses is essential: “Documents in the same cluster behave similarly with respect to relevance to information needs.” We are not trying to do *K*-Clustering, where we have a given number of *K* clusters. Thus we define the document clustering as follows:

Given a similarity function for the Document Space *D* as $sim:\; D\times D\rightarrow \mathbb {R}^{+}$ and an $\epsilon \in \mathbb {R}^{+}$. We search for a minimal number of clusters, so that every two documents *x*,*y* in one cluster have *sim*(*x*,*y*)≥*ε*. We will use this approach as Definition 1. For technical terms we refer to [5].

A *hard clustering* defines that every document belongs to only one cluster, whereas *soft clustering* allows documents to belong even to one or more clusters with a distinct probability. We will introduce a novel graph structure that can also handle soft clustering.

This paper uses a novel reformulation of document clustering as a graph partition problem to get new insights to the problem itself. We hope that this leads to new heuristics and a deeper understanding of the problem. We will first discuss related work and the current state of the art and point out, why a graph-theoretical approach is novel. Thus, after considering some preliminaries we will introduce pseudostable sets and pseudocliques, which are deeply related to graph coloring and stable sets. We will reformulate soft document clustering as a graph problem, where we seek a minimal partition in pseudeostable sets. After introducing a greedy and integer linear programming approach we will make a proof of concept on some real world data.

### Related work and state of the art

Recent research has focused on methods and heuristics to solve document clustering. The authors of [6] for example tried to cluster documents received from MEDLINE database using evolutionary algorithms, whereas [7] used machine learning approaches, see also the work of [8]. As mentioned previously, only a few authors like [9] mentioned graphs. As [10] points out, unfortunately “no single definition of a cluster in graphs is universally accepted, and the variants used in the literature are numerous”.

There has also been a lot of research which is related, but had a different scope. The authors of [11] for example discussed document clustering in the context of search queries, whereas [12] discussed the topic of hierarchical clustering. In the field of bioinformatics or life science informatics, the automatic classification and recognition of texts according to their medical, chemical or biological entities is in the scope of researchers (see [13], [14] or [15]). Document Clustering has been in the focus of research for the last decades and interest is steadily growing. This gets also obvious when observing the increasing number of competitions in this field, for example TREC – Text REtrieval Conference –, see [16].

Using a Graph Partition for clustering has been widely discussed in literature. Schaeffer points out that “the field of graph clustering has grown quite popular and the number of published proposals for clustering algorithms as well as reported applications is high” [10]. Usually directed or weighted graphs are subject of research. However, we would like to emphasize that for problem complexity reasons it is suitable to focus on simple graphs. The work reported in [17] explains that a graph partition in cliques or stable sets is most common.

We can conclude that focusing on graph clustering only is a novel approach and the generalization of soft document clustering introduced in [1] leads to the conclusion that we can focus on the graph-theoretical toolbox to get new insights on document clustering – or clustering in general.

### Preliminaries

#### Document clustering

First of all, with Definition 1 we gain a starting point covering hard document clustering. We will suggest a slightly different approach to cover both hard as well as soft clustering.

##### **Definition 1**

(Hard Document Clustering) Given a set of documents *D*={*d*_1_,…,*d*_*N*_} and a similarity measure $sim:\; D\times D\rightarrow \mathbb {R}^{+}$ as well as a bound $\epsilon \in \mathbb {R}^{+}$. We search for a minimal number of clusters, so that for every two documents *x*,*y* sharing the same cluster *sim*(*x*,*y*)≥*ε* holds.

A graph partition into stable sets or cliques can be generalized to be universal in such a way that it can handle hard clustering as well as soft clustering:

##### **Definition 2**

(Soft Document Clustering, according to [5]) Given a set of documents *D*={*d*_1_,…,*d*_*N*_} and a similarity measure $sim:\; D\times D\rightarrow \mathbb {R}^{+}$ as well as two bounds $\epsilon, \iota \in \mathbb {R}^{+}$ and *ι*<*ε*. We search for a minimal number of clusters, so that for every two documents *x*,*y* sharing the same cluster *sim*(*x*,*y*)≥*ι* holds and two documents *x*,*y* with *ε*≥*sim*(*x*,*y*)≥*ι* may share multiple clusters *a*,*b* if two documents *x*^′^,*y*^′^ within cluster *a*,*b* exist so that *sim*(*x*^′^,*x*)≥*ι* and *sim*(*y*^′^,*y*)≥*ι*.

We argue that a simple graph for a representation of documents for the purpose of document clustering is not a limitation. The graph does not need to be directed, since for two documents *d*_*i*_,*d*_*j*_, *sim*(*d*_*i*_,*d*_*j*_)=*sim*(*d*_*j*_,*d*_*i*_) always holds. Since every clustering algorithm needs to decide, if two documents are in one cluster, there is no need to assign a weight to the edge. If a previous measurement algorithm decides that two documents cannot be in the same cluster, the value should be set that way that there is an edge.

### Graph theory

Given a Graph *G*=(*V*,*E*) with nodes or vertices in a set *V* and a set of edges *E*. Two nodes *u*,*v*∈*V* are adjacent, if an edge (*u*,*v*)∈*E* exists. The graph coloring problem is to assign a color to each node so that every two nodes that are adjacent have a different color. The minimal number of colors needed to color a graph is called chromatic number and denoted with *χ*(*G*). This problem has many applications and has been studied extensively. It is on most graphs $\mathcal {NP}$-complete, see [18].

For every feasible coloring of *G* all nodes sharing the same color imply a stable set in *G*. *S* is a *stable set* in *G* if (*u*,*v*)∉*E* ∀*u*,*v*∈*S*. Thus we have a partition of *G* into stable sets. It is anyhow possible to use a set covering approach, where the set of vertices has to be covered by a minimum number of stable sets, see [19]. This is very useful in the context of linear programming. As Hansen et al. mentioned, this approach involves an exponential number of variables, making the problem complex. Many optimization problems on graphs can be formulated as set covering problems.

### Steps to realize clustering

We will discuss the steps to realize a document clustering in order to point out, which parts can be done by this novel approach. To get an overview about the necessary steps we will follow Jain et al. in [4, 266f]):

1. *Pattern representation*: read structures and information (feature extraction and selection) and find a feasible representation for the documents.
2. Define a *similarity measure* appropriate to the data or document domain.
3. The *clustering* or grouping process.
4. Optional: *data abstraction*, which means for example to make the cluster human-readable.
5. Optional: *assessment of output*, which is the process of validating the results.

The last two steps are only relevant for the application part. However, we will see that this can also be very easily realized with this novel approach.

We suppose that we have a suitable pattern representation of our data. We will discuss some similarity measures as well in the next section, but we need to point out that this leads to different issues not related to the clustering process itself. The evaluation of similarity measures is very complex, which has been described in the work of Milligan [20], who tried to evaluate the error given by different similarity measures by dividing between external and internal criterions. Huang stated that the quality of a clustering result usually is evaluated using the two evaluation measures purity and entropy [21]. Thus, we will suppose that we have a given similarity measure. For evaluating errors according to the similarity measure we would need an additional gold standard to measure purity and entropy.

Thus we only need to discuss in detail how PS-Document Clustering can achieve the clustering process and how data abstraction and eventually the validation can be done.

#### Similarity measures

There is a lot of work focusing on similarity measures for documents in a document space $\mathbb {D}$. All of them use characteristics of documents, so-called *features*, and map them to a real number. A very common approach is to use a *vector space model*. Here, all documents in $\mathbb {D}$ are vectors in a so-called *Feature Space*$\mathbb {D}^{N}$. Thus, the distance between vectors can be calculated, see [2, 84f]. Following [22, 275], it is possible to use a weighted vector of words, using stop words and use the combination of *term frequency* and *inverse document frequency* as TF.IDF measure (*term frequency–inverse document frequency*). This is one of the most important measures, see [23]. For *N* documents in $\mathbb {D}$ the computation can be done following [24, 8f]: *f*_*ij*_ is the occurence of the word *i* in document *j*. Term frequency *TF*_*ij*_ is computed after normalization on [ 0,1]: $TF_{ij}=\frac {f_{ij}}{\max _{k} f_{kj} }$. Computing the inverse document frequency can be done with $IDF_{i}=\log _{2} \left (\frac {N}{n_{i}}\right)$. Here, *n*_*i*_ is the occurrence of word *i* in all *N* documents. The TF.IDF measure is not given by *TF*_*ij*_×*IDF*_*i*_. This leads to a vector in $\mathbb {R}$ for every document and we can calculate the vector distance for two documents by using cosinus-distance, the euclidian norm or other norms.

Therefore, a first approach can be done using a distance model *d*_*V*_ based on the vector of weighted words using NLP techniques for the abstracts. In addition a distance according to the journal, which is *d*_*J*_(*x*,*y*)={0,1}. Thus we have

$$d_{1} (x,y)= \frac{d_{V}(x,y)+d_{J}(x,y)}{2}$$

The second approach is the usage of *d*_2_=*d*_*V*_. The third approach uses only the Tanimoto similarity on keywords (MeSH terms, see “Results” section) *d*_3_=*sim*. We may use them to calculate the Tanimoto similarity, also known as Jaccard similarity

$$sim(a,b)= \frac{|M_{a} \cap M_{b}|}{|M_{a} \cup M_{b}|} \; \forall a,b\in D$$

with *sim*:*M*×*M*→[ 0,1]. As we will show in the next section, this first approach is not suitable for all applications.

## Methods

### Pseudostable sets and Pseudocliques

We will now discuss novel graph structures. Pseudostable sets were first introduced in [25] as a graph partition problem in the context of the Train Marshalling Problem covering the rearrangement of cars of an incoming train in a hump yard. They are still under research in several contexts. In this paper we will apply pseudostable sets in a completely new context and also introduce pseudocliques and the corresponding graph covering problem. Thus, the whole approach presented in this paper is novel.

We consider a simple Graph *G*=(*V*,*E*) with a subgraph *B*⊂*G* of so-called blue nodes and edges. *B* can be chosen absolutely arbitrary. For example, it is also possible that *B*=*∅* or *B*=*G*.

#### A set-covering approach

At first we need to define two different subsets of the graph *G* to create a set covering:

##### **Definition 3**

[Pseudostable Tuple] *T*⊂*G* is a *pseudostable Tuple*, if it is the union of two stable sets *D*_1_ and *D*_2_ and a path *p* such that

$$T = D_{1} \cup p \cup D_{2} $$

The intersection of *D*_1_ and *p* as well as *p* and *D*_2_ consist of one node. The set *p* is pairwise disjunct and consists of three nodes and two edges in *B*. That means, *p*_*j*_⊂*B*(*G*), |*V*(*p*_*j*_)|=3 and *p*_*j*_ is connected and circle-free. *T* can also be stable if *D*_1_ is stable and *p*=*D*_2_=*∅*. Then the value of *T* is *ζ*(*T*)=1, otherwise *ζ*(*T*)=2.

**Fig. 1:**
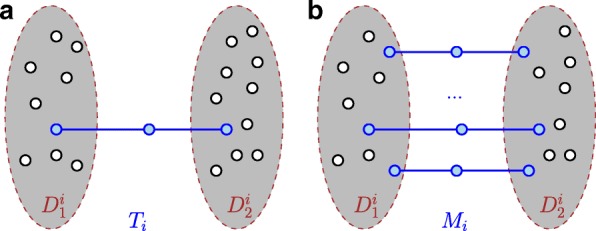
A pseudostable tuple *T*_*i*_ in (**a**) and a multiple pseudostable tuple *M*_*i*_ in (**b**). Both sets *D*_1_ and *D*_2_ are stable and some blue paths of length 3 exist between both. The sets $\mathfrak {P}(T_{i})$ and $\mathfrak {P}(M_{i})$ consist of all blue nodes which are neither in *D*_1_ nor in *D*_2_

It is also possible to allow more than one path between *D*_1_ and *D*_2_, see Fig. 1 for an illustration.

##### **Definition 4**

(Multiple pseudostable Tuple) *M*⊂*G* is a *Multiple pseudostable Tuple*, if it is the union of two stable sets *D*_1_ and *D*_2_ and paths *p*_1_,…,*p*_*i*_ such that

$$M = D_{1} \cup p_{1} \cup \ldots \cup p_{i} \cup D_{2} $$

The intersection of *D*_1_ and *p*_*j*_ as well as *p*_*j*_ and *D*_2_ (*j*∈{1,…,*i*} consists of one node. The sets *p*_*i*_ are pairwise disjunct and consist of three nodes and two edges in *B*. That means, *p*_*j*_⊂*B*(*G*), |*V*(*p*_*j*_)|=3 and *p*_*j*_ connected and circle-free. *T* can also be stable if *D*_1_ is stable and *i*=0 and *D*_2_=*∅*. Then the value of *T* is *ζ*(*M*)=1, otherwise *ζ*(*M*)=2.

Since we usually have more than one *M* or *T* we will use indices to denote them. In the following, *M*_*i*_ or *T*_*i*_ are an arbitrarily chosen *M* or *T*. We denote for *M*_*i*_ or *T*_*i*_ both stable sets with $D^{i}_{1}$ or $D^{i}_{2}$.

It is possible that $D^{i}_{2}=\emptyset $, but it is always $D^{i}_{1}\neq \emptyset $. We define that *Pf*(*T*) or *Pf*(*M*) is the union of all paths in *T* or *M*. *Pf*(*T*_*i*_)=*∅* or *Pf*(*M*_*i*_)=*∅* if, and only if $D^{i}_{2}=\emptyset $. Every pseudostable Tuple is a multiple pseudostable Tuple. We usually search for a minimal set cover *S* of *G* with *S*={*T*_1_,…,*T*_*n*_} or *S*={*M*_1_,…,*M*_*n*_}. We define the weight *w* as

1 $$ w(S) = \sum_{i=1}^{n} \zeta(S_{i}) + \sum_{i=1}^{n} \sum_{j\in \{ 1,\ldots,n\}\setminus \{i\}} w_{i,j}  $$

$$w_{i,j}=\left\{\begin{array}{ll} -1 & M_{i}\cap S_{j}=D_{1}^{i}=D_{2}^{j}\\ 0 & otherwise \end{array}\right.$$

The first condition ensures that two stable sets *D* in two individual, but identical tuples are not weighted two times. All other cases can be ignored. This weight holds for multiple pseudostable tuples as well as pseudostable tuples. With a weight we can define a minimization problem.

For a given Graph *G*=(*V*,*E*) with a blue subgraph *B*⊂*G* we define $\mathfrak {T}=\{T_{1},\ldots,T_{n}\}$ as the subset of all pseudostable tuples in *G* with *B*.

With $\mathfrak {P}(T)$ we denote all inner nodes of paths within *T*, which means

$$\mathfrak{P}(T_{i}) = T_{i} \setminus\{ D^{i}_{1} \cup D^{i}_{2}\}$$

Or, it is also possible to define it according to *Pf*(*T*_*i*_) as $Pf(T_{i}) \setminus \{ D^{i}_{1} \cup D^{i}_{2}\}$ which is the same.

The Definition of the optimization problem can now be written as:

2 $$ \begin{array}{lll} \text{minimize} & \sum\limits_{i=1}^{n} t_{i} \zeta(T_{i}) + & \sum\limits_{i=1}^{n} t_{i} \sum\limits_{j=1}^{n} t_{j} w_{i,j} \\ \text{subject to}& \sum\limits_{T \in\mathfrak{T}: v\in Pf(T)} t_{i} & =1, \forall v\in V \\ &\sum\limits_{T \in\mathfrak{T}: v\in T} t_{i} & \geq 1, \forall v\in V \\ & & t_{i} \in \{0,1\} \end{array}  $$

The variable *t*_*i*_ indicates, if set *T*_*i*_ is chosen for this set covering. The minimization term refers to the weight given in Eq. 1. The next line ensures that every node *v*∈*V* is assigned to exactly one node within a path of a pseudostable tuple. The last condition ensures that every node *v*∈*V* is covered by at least one set.

If we want to allow intersections between inner nodes of paths *p* we can simply skip the second condition. Thus, our minimization problem is as follows:

3 $$ \begin{array}{lll} \text{minimize} & \sum\limits_{i=1}^{n} t_{i} \zeta(T_{i}) + & \sum\limits_{i=1}^{n} t_{i} \sum\limits_{j=1}^{n} t_{j} w_{i,j} \\ \text{subject to} &\sum\limits_{T \in\mathfrak{T}: v\in T} t_{i} & \geq 1, \forall v\in V \\ & & t_{i} \in \{0,1\} \end{array}  $$

Both 2 and 3 hold for pseudostable tuples *T* as well as multiple pseudostable tuples *M*.

A set covering of a graph *G*=(*V*,*E*) with a subset *B*⊂*G* of blue nodes and edges with a set of *T* or *M* also induces the Graph of this set covering. In this graph, every stable set *D* within the covering of *G* induces a node and every path an edge:

##### **Definition 5**

(Graph of a set covering) Given a set covering *S*={*S*_1_,…,*S*_*n*_} of a graph *G*=(*V*,*E*) with a subset *B*⊂*G* of blue nodes and edges with pseudostable tuples *T*_1_,…,*T*_*n*_ or multiple pseudostable tupels *M*_1_,…,*M*_*n*_. Then we define *G*_*S*_=(*V*,*E*) as the Graph of the set covering with

$$V = \{ D \subset S_{1},\ldots,S_{n}\}$$

$$E = \{ (D^{i}_{1}, D^{i}_{2}) \; i\in\{1,\ldots,n\}\;\text{if}\; D^{i}_{2}\neq\emptyset\}$$

Now we can define the minimization problem as follows. We will continue using the naming introduced in [25].

##### **Definition 6**

(minPS) We search for a minimal set covering *S* of the graph *G*=(*V*,*E*) with a subset *B*⊂*G* of blue nodes and edges with pseudostable tuples *T* according to *2*, where *G*_*s*_ is acyclic and *δ*(*v*)∈{0,1,2} for all *v*∈*V*(*G*_*S*_).

##### **Definition 7**

(minMPS) We search for a minimal set covering *S* of the graph *G*=(*V*,*E*) with a subset *B*⊂*G* of blue nodes and edges with multiple pseudostable tuples *M* according to *2*, where *G*_*s*_ is acyclic and *δ*(*v*)∈{0,1,2} for all *v*∈*V*(*G*_*S*_).

We denote minPS’ and minMPS’ as the corresponding minimization problem according to 3. minPS-a and minMPS-a are the corresponding minimization problems without restrictions on the graph *G*_*S*_. This means

##### **Definition 8**

(minPS’-a) We search for a minimal set covering *S* of the graph *G*=(*V*,*E*) with a subset *B*⊂*G* of blue nodes and edges with pseudostable tuples *T* according to *3*.

##### **Definition 9**

(minMPS’-a) We search for a minimal set covering *S* of the graph *G*=(*V*,*E*) with a subset *B*⊂*G* of blue nodes and edges with multiple pseudostable tuples *M* according to *3*.

Now we have a definition as set covering problem. This is also useful to proof the $\mathcal {NP}$-completeness of this problem. Now we will make a definition using a graph partition approach.

The formulation of minPS or minMPS as graph partition problem is very clear and concrete, but it gets unhandy when handling the variants minMPS-a or minMPS’. In [1] we showed that our new approach using set covering is equivalent to the work described in [25] and thus also a graph partition approach. Every graph covering leads to a graph partition.

### A new clustering approach with pseudostable sets

We will now create a Graph *G*=(*V*,*E*). Every document in our document set is a node *n*∈*V*. We would like to follow Schaeffer [10] and restrict our similarity measure on [0,1], “where one corresponds to a ’full’ edge, intermediate values to ’partial’ edges, and zero to there being no edge between two vertices.” Now we can define a limit and define edges between nodes if they are not similar enough.

Given a set of documents *D*={*d*_1_,…,*d*_*N*_}, a similarity measure

$$sim:\; D\times D\rightarrow \mathbb{R}^{+}$$

and an $\epsilon \in \mathbb {R}^{+}$. The function is limited to [ 0,1]. If not, we normalize it as *sim*^′^: *D*×*D*→[ 0,1] as

$$sim'(x,y)= \frac{sim(x,y)}{\max sim(x,y)}$$

Our graph *G* is now defined as

$$G = (V, E) \; V=D \; $$

$$E = \{ (d_{i}, d_{j}) \; | \, sim(d_{i}, d_{j}) \leq \epsilon \} $$

Edges between documents exist only if they are less similar than *ε*. A graph coloring approach would now create a graph partition into stable sets. This would result in a hard clustering. To achieve a soft clustering we can define another bound *ι* with 0<*ι*<*ε* and another set of edges *B*=(*V*,*E*^′^) with

$$ {E^{\prime}} = \left\{ \left(d_{i}, d_{j}\right) \; | \, \iota \leq sim(d_{i}, d_{j}) \leq \epsilon \right\} $$

We can see that *B*⊂*G*. We have two kinds of edges: on the one hand those edges *e*⊂*G* but not in *B*. We call them black. These refer to documents which are not similar. On the other hand, those edges *e*⊂*B* called blue refer to documents that are also not similar, but less not similar then those edges not in *B*. If we set *ι*=*ε* then *B*=*∅* and we have a hard clustering. If *B*≠*∅* we have a soft clustering if we use the following definition:

#### **Definition 10**

(PS-Document Clustering) Given a graph *G* with *B*⊂*G* according to the definition above. A solution of minMPS’-a gives a Document Clustering in multiple pseudostable sets with *ζ*(*G*) Cluster and Documents that are in between those clusters *D*.

Before continuing, we will create the weighted Graph of the clustering. This definition is highly related to Definition 5. Every node refers to a document cluster and every edge refers to the number of paths between both clusters.

#### **Definition 11**

The *weighted Graph of the Clustering* is a Graph *G*_*c*_=(*V*_*c*_,*E*_*c*_) with

$$V_{c} = \left\{ D^{i}_{j} \in P_{i} \right\},\,d\left(D^{i}_{j}\right)=\left|D^{i}_{j}\right| $$

$$E_{c} = \left\{\left(D^{i}_{j}, D^{i}_{k}\right),d\left(D^{i}_{j}, D^{i}_{k}\right)>0\right\} $$

The weight $d\left (D^{i}_{j}, D^{i}_{k}\right)$ can be defined in multiple ways. The easiest way is to sum all paths between both stable sets:

$$\begin{aligned} d_{s}\left(D^{i}_{j}, D^{i}_{k}\right) &= |P| \, \text{with}\,\\ P &=\{ p \,|\, p \cap D^{i}_{j} \neq \emptyset \,\text{and}\, p\cap D^{i}_{k} \neq \emptyset\} \end{aligned} $$

but more intuitive is the following weight:

$$\begin{aligned} d\left(D^{i}_{j}, D^{i}_{k}\right) &= \sum_{p} \frac{|N(v)\cap D^{i}_{j}| + |N(v)\cap D^{i}_{k}|}{|D^{i}_{j}|+|D^{i}_{k}|} / |p|\\ \forall p&=(u,v,w) \,\text{with}\, p\cap D^{i}_{j} \neq \emptyset \,\text{and}\, p\cap D^{i}_{k} \neq \emptyset \end{aligned} $$

This weight counts all inner nodes *v* within a path *p*=(*u*,*v*,*w*) the number of neighbours in one of the stable sets. We can use this as a measure for the similarity of this node with the given stable set. If there is no edge from *u* to one node in the set, it might also be assigned to that stable set. Each such edge decreases this possibility. We normalize with the number of paths and thus have a value in between [ 0,1].

#### **Example 1**

**Fig. 2:**
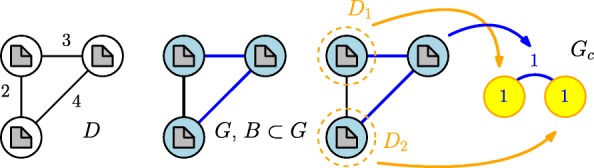
Figure explaining the Example 1. It illustrates the documents *D* with their similarity, the resulting Graph *G*, its partition into pseudostable sets *D*_1_, *D*_2_ and the weighted graph *G*_*C*_ of that clustering

Given three documents with some similarity, see Fig. *2*. We set *ι*=2,5 and *ε*=5. We obtain a graph with blue nodes and two blue edges. One edge is black. If we partition into pseudostable sets, we find two clusters with one document and one document in between both. The weighted graph of this clustering is also shown in Fig. *2*. Every cluster is associated with a node in *G*_*c*_.

If we precisely use the Definition of pseudostable sets given by graph partition approach, this Graph needs to be acyclic. However, we will follow the definition given in the first chapter and just notice that the definition by set covering approach is more clear. This Graph is important for visualization and assessment.

### New approaches

The main problem is that minMPS’-a is $\mathcal {NP}$-complete. First of all, we will describe an Integer Linear Programming approach for calculating optimal solutions. Afterwards, we will discuss our Greedy-Approach for solving minMPS’-a. We want to show a small example on how all approaches solve the problem. Finally, we will discuss the application on some real-world data and the output.

#### Integer linear program

Given a graph *G*=(*V*,*E*) with a subset *B*⊂*G* of blue nodes and edges. *T* is the list of all paths with length three within *B*.

*y*_*k*_ denotes the variable, which indicates that a color *k* is used. Is *y*_*k*_=0 color *k* will not be used. *x*_*i*,*k*_ indicates, if a node *i*∈*G* is colored with color *k*. Color *k*=0 will be used for those nodes which are in a path *p*.

$$\begin{array}{*{20}l} \text{[minMPS'-a-IP]}\quad \text{min} \quad \sum_{k=1}^{n} y_{k}& \end{array} $$

4 $$\begin{array}{*{20}l} \sum_{k=1}^{n} x_{i,k} = 1 & \qquad \qquad \qquad\qquad\qquad\forall i=0,\ldots,n \end{array} $$

5 $$\begin{array}{*{20}l} x_{i,k} -y_{k} \leq 0 & \qquad \qquad\forall i=0,\ldots,n, \forall k=1,\ldots,n \end{array} $$

6 $$\begin{array}{*{20}l} x_{i,k} +x_{j,k} \leq 1 & \qquad\qquad(i,j)\in E(G), \forall k=1,\ldots,n \end{array} $$

7 $$\begin{array}{*{20}l} x_{i,0} \leq 0 & \qquad\qquad \qquad\qquad\qquad \quad\forall i \not\in B(G) \end{array} $$

8 $$\begin{array}{*{20}l} x_{i,k} \geq 0 & \end{array} $$

9 $$\begin{array}{*{20}l} y_{k} \leq 1 & \end{array} $$

10 $$\begin{array}{*{20}l} x_{i,k}+x_{j,k}+x_{v,0}-2 \leq 0 & \qquad\qquad(i,v,j)\in T, \forall k=1,\ldots,n \end{array} $$

11 $$\begin{array}{*{20}l} x_{i,0}+x_{j,0}+x_{v,0} \leq 1 & \qquad\qquad(i,v,j)\in T, \forall k=1,\ldots,n \end{array} $$

$$\begin{array}{*{20}l} &x_{i,k},y_{k}\in\mathbb{Z} & \end{array} $$

Condition 4 ensures that every node has a color or color *k*=0. For each node *i* and every color *k* *x*_*i*,*k*_−*y*_*k*_≤0 is necessary. Is node *i* not in color *k*, inequality 6 holds. But if it is in color *k*, *y*_*k*_=1 and thus the inequality holds. Two connected nodes *i*,*j* must not share the same color *k*>0. Thus *x*_*i*,*k*_+*x*_*j*,*k*_≤1, see condition 7. Condition 8 ensures that no node which is not within *B* can be assigned to color *k*=0. The last conditions ensure that if a node *v* is within color *k*=0 all within *B* connected nodes to *v* have a different color.

In practise we can only apply minMPS’-a-IP to small instances due to the exponential runtime.

#### Greedy-approach

Given a graph *G*=(*V*,*E*) with a subset *B*⊂*G* of blue nodes and edges. We run on a (not necessary minimal) graph coloring *f*:*V*→*F* with $F\subset \mathbb {N}$ and implement a greedy algorithm that puts every possible path in between two stable sets. Since we do not have perfect graphs for documents clustering we need to use heuristics to get an approximate graph coloring. Alternatively we can use the complement graph $\overline {G}$ and use a partition into cliques which results in a coloring of *G*.

We will iteratively try to eliminate stable sets *D* given by the graph coloring heuristic and thus use the properties and characterizations of pseudostable sets:

- For each color *i* we consider node *u* in it:
  - Is this node not an endpoint of a path *p* (which ist stored in *ende*) check if there exist two nodes *v*,*w*∈*G* which are connected by blue nodes with *u* and are in different color classes.
  - Is this true, remove *u* from *i* and create a new path *p*= [ *v*,*u*,*w*].

See algorithm 1 for pseudo code. We can not give an approximation guarantee and we will show that this heuristic does usually not provide an optimal solution.


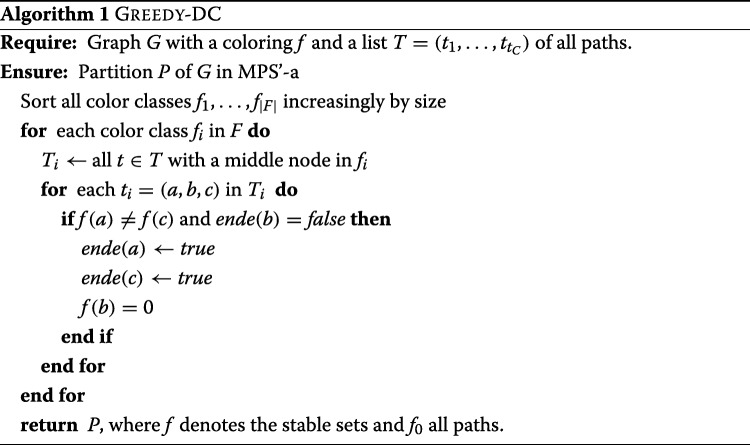


We have used the following heuristics to start the graph coloring:

- Coloring using the *greedy independent sets* (GIS) approach with a runtime in *O*(*mn*), see [26].
- Coloring using the SLF Approach with a linear runtime *O*((*m*+*n*) log*n*) (see [26] and [27]).
- Clique Partition on $\overline {G}$ using the TSENG clique-partitioning algorithm described in [28] with a worst case runtime *O*(*n*^3^).

We assume to get a better solution by the third approach for instances where we have a huge amount of edges. Here, it might be less complex to solve the clique partition problem on the complement graph.

We will generate some random instances using the model of Gilbert, see [29]. This creates a simple undirected graph *G*=(*V*,*E*) with (*n*(*n*−1))/2 possible edges as a model . Edges will be added with probability 0<*p*<1.

Erdös and Rényi designed a similar approach , were all Graphs with exactly *n* nodes and 0<*m*<(*n*(*n*−1))/2 edges are equal probable, see [30].

Both algorithms have a quadratic runtime. For small *p*, Batagel and Brandes described a linear time approach with a runtime in *O*(*n*+*m*), where *m* is the number of created edges, see [31].

We will chose *p*=0.75 and a second probability *p*^′^=0.2, which decides whether edges are colored blue. This refers to the instances we have seen on real-world data.

**Fig. 3:**
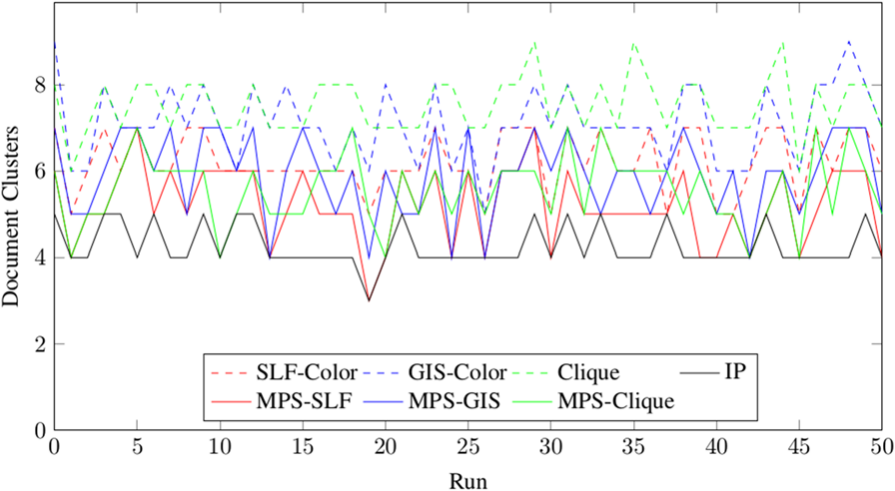
Results for random instances with *n*=15 nodes. The dotted plots show the upper bounds computed with the graph coloring heuristics SLF, GIS and Clique. The continues plots show the results of the Greedy Approach. In addition the solution computed with the integer linear program is shown

**Fig. 4:**
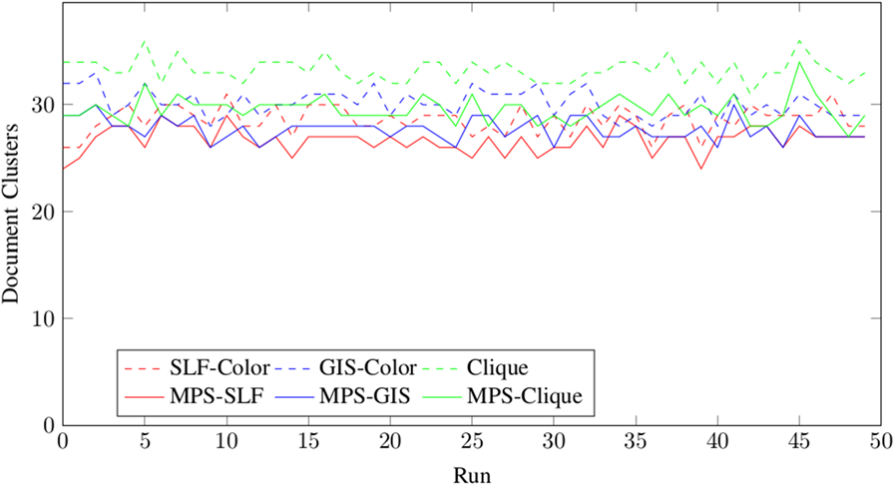
Results for random instances with *n*=100 nodes. The dotted plots show the upper bounds computed with the graph coloring heuristics SLF, GIS and Clique. The continues plots show the results of the Greedy Approach

We will show the results for different random instances with 15 nodes in Fig. 3 and with 100 nodes in Fig. 4. We have also added the results of the integer linear program for small instances.

As we can see in both figures, the clique approach gives the worst partition into stable sets for large instances, but the greedy approach eliminates most stable sets. SLF gives in general better results than GIS and also has a better runtime.

#### A parallel approach using divide and conquer for generating *G*

Since the computation of *G* obviously consists of independent steps when computing the similarity *sim*, this time-consuming step can be easily parallelized. A lot of research has already focused on the parallelization of data mining approaches, see [32] and [33]. Many problems can be naturally be expressed with the divide and conquer pattern of parallelisation, in particular every time when the solution to a problem can be found by dividing the set into subproblems which can in turn be solved separately. Afterwards the solutions can be merged to a global solutions. This is exactly what can be assigned to our problem: We can divide the input set into small subsets, compute each separately and afterwards merge the solutions. We can expect a high speed up for large instances by using this approach, see [34].

We have thus created methods to save and load instances and to append saved instances *G*_2_ to an already loaded instance *G*_1_. This calculates all missing edges in *G*_1_∪*G*_2_. We currently utilize the parallel execution of system threads using BASH scripts, but it is easy to adopt the scripts to use job schedulers like SLURM, SGE and so on.


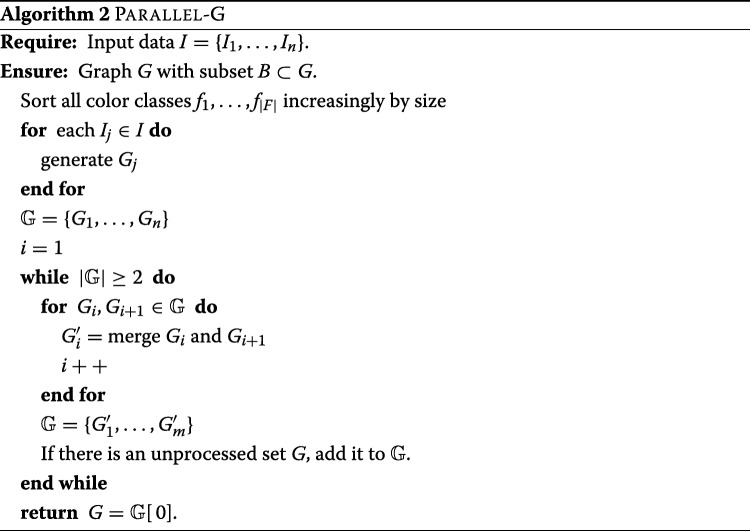


See algorithm 2 for an example of how to adapt the divide and conquer approach to the generation of the graph *G*.

### Computing the bounds *ε* and *ι*

Despite the time complexity of generating the Graph *G* out of an input instance, another yet not discussed topic is the computation of the two bounds *ε* and *ι*. We suggest two different approaches. One relies on external criteria: We will choose both bounds in such a way that we can estimate the number of clusters. The second approach relies on internal criteria: If we have a set of pairs of documents {(*d*_*i*_,*d*_*j*_),…,(*d*^′^_*i*_,*d*^′^_*j*_)} and expect them to be in different clusters, we can approximate *ε* and *ι*.

#### External criteria: bounds for the number of clusters

Since the coloring number *χ*(*G*) of an Graph *G* is an upper bound for *ζ*(*G*), we can try to use an upper bound for graph coloring for a graph obtained for given values *ε* and *ι* to approximate these values. Given that we want *n* clusters. We choose *ε* and *ι* and calculate an upper bound *u*=*u*_*χ*_(*G*). If *u*>*n* or *u*<*n* we can adjust *ε* and *ι* according to those values.

Following [28] we find an upper bound for $\overline {\chi }(G)$:

$$\overline{\chi}(G) < \left\lfloor \frac{1+\sqrt{4n^{2}-4n-8e+1}}{2}\right\rfloor $$

where *n* is the number of nodes and *e* the number of edges in *G*. The number of nodes in *G* and $\overline {G}$ is the same. The number of edges $\overline {e}$ in $\overline {G}$ is *n*^2^−*e*. Thus it follows that

$$\begin{aligned} \chi(G) = \overline{\chi}(\overline{G}) &< \left\lfloor \frac{1+\sqrt{4n^{2}-4n-8(n^{2}-e) +1}}{2}\right\rfloor \\ &= \left\lfloor \frac{1+\sqrt{-4n^{2}-4n+8e +1}}{2}\right\rfloor \end{aligned} $$

But we have to conclude that “‘[i]n particular, it appears that our algorithms perform increasingly better relative to the coloring algorithms for larger graphs.”’ [28, 11]. Thus this bound is only feasible for dense and large graphs. Other approaches according to the complementary graph can be found in the works of Feder and Motwani [35], Gramm et al. [36] and Benati et al. [37].

Since there is no trivial and easy upper bound for graph coloring, we have to use heuristics and algorithms to solve this issue. See “Results” section for results.

#### Internal criteria: approximate *ε* and *ι*

Given a set of pairs of documents *P*={*p*_1_,…,*p*_*n*_} with *p*_*i*_=(*d*_*i*_,*d*^′^_*i*_) and $d_{i}\in \mathbb {D}$ and we expect each pair of documents to be assigned to different clusters. Then we can set

$$\epsilon < \max_{p_{i},\ldots,p_{n}} sim(d_{i},d'_{i})$$

This leads to blue edges at least between all pairs of documents in *P*. Thus, all these documents are not in one cluster.

## Results

We apply this new approach to perform document clustering over some subsets of MEDLINE data. MEDLINE (Medical Literature Analysis and Retrieval System Online) is a bibliographic database maintained by the National Center for Biotechnology Information and covers a large number of scientific publications from medicine, psychology, and the health care system. For the clustering use case, we study MEDLINE abstracts and associated metadata that are processed by ProMiner, a named entity recognition system ([38]), and indexed by the semantic information retrieval platform SCAIView ([39]). SCAIView also offers an API that allows programmatic access to the data. Currently, we only use meta information like title, journal, publishing year and the so-called MeSH terms for our experiments.

We extract subset *D* of MEDLINE documents from SCAIView. Every document on MEDLINE should have a list *M* of keywords, the MeSH terms. We may use them to calculate the Tanimoto similarity, also known as Jaccard similarity. This first approach is not suitable for all applications as we will show in the next section. This is why we postulate a distance model based on the vector of weighted words using NLP techniques.

We then build a graph *G* according to the bounds *ε* and *ι*. Following this, we create the directed graph of that partition by applying the Greedy approach. We also store further metadata like year and publishing journal of documents in nodes and edges.

We will now describe the result of one input set given by [14] and discussed by [15]. In both publications the first dataset consisted of 1660 documents obtained from two different queries ’escherichia AND pili’ and ’cerevisiae AND cdc*’. Both returned the same number of 830 documents. We had a similar result with 1628 documents trying to reproduce this query with data till the year 2001. This dataset covers two different topics, whereas the second dataset is related to the developmental axes of Drosophila. We will now discuss several outputs of our new approach.

Consequently, we have *n*=1628 nodes (documents). The number of edges *e* and blue edges *b* depend on the different values of *ι* and *ε* and the priorly used approach for similarity. We will discuss the following three measures: First an approach using a distance model *d*_*V*_ based on the vector of weighted words using NLP techniques for the abstracts following “Similarity measures” section. In addition a distance according to the journal, which is *d*_*J*_(*x*,*y*)={0,1}. Thus we have

$$d_{1} (x,y)= \frac{d_{V}(x,y)+d_{J}(x,y)}{2}$$

The second approach is the usage of *d*_2_=*d*_*V*_. The third approach uses only the Tanimoto similarity on MeSH terms described above, thus *d*_3_=*sim*.

We wanted to compare our results with those given by [14] and [15]. We will show that the comparability of clusterings with previous studies is highly dependent on the choice of this distance measurement. Every clustering produces different details with the same heuristic running in the background. Thus it is now not totally clear to connect clusters to topics. But first of all we want to proof our new approach and reproduce the results of both [14] and [15] which we will discuss for every distance measure.

**Fig. 5:**
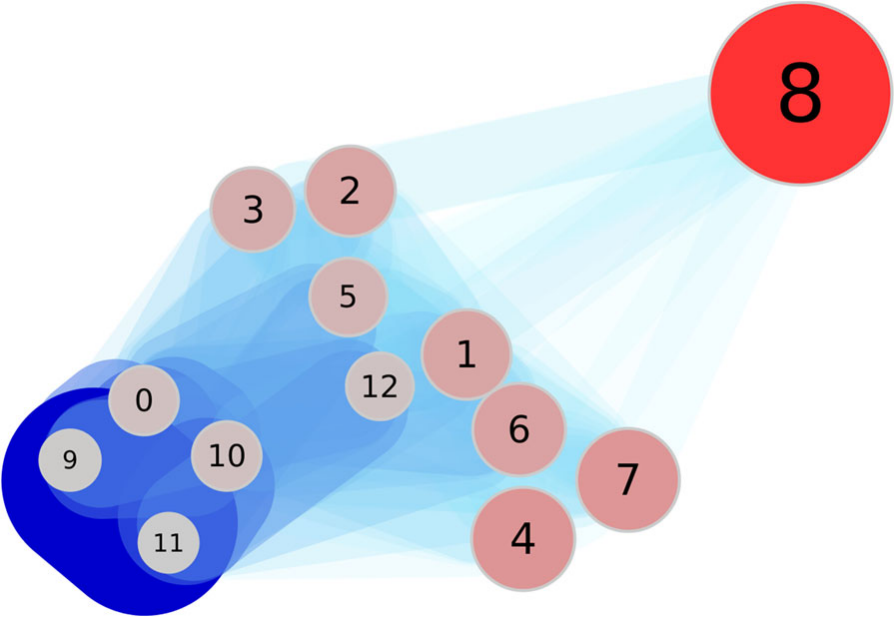
The partition of the first dataset with distance *d*_1_. The numbers identify the clusters. The size of a node is related to the number of documents included. The edges and their widths and color describe their weight. A darker blue edge has a greater weight

**Distance measure*d***_**1**_: The results of our clustering approach with distance measure *d*_1_ are shown in Fig. 5. We got 13 clusters (Cluster 0 to 12), containing between 5 (Cluster 11) and 359 (Cluster 8) documents each.

Our clustering heuristic is able to produce clusterings of variable detail by choosing different values for *ι* and *ε*. We have chosen values that visualize the benefit of the new graph-theoretical approach. Referring to Fig. 5 it is easy to see that the first cluster is given by cluster 8. It has only weak dependencies and relations to other clusters as can be seen by the edges in the graph. Clusters 0, 9, 10, 11 are highly dependent and thus form the second cluster agglomeration. We can see a similar result to [15]: the terms of both clusters describe the general concepts that are relevant to both search queries. So our approach produces similar results with this distance measure.

Those clusters which are in between the two main clusters share topics with both. For example cluster 7 is related to ‘Molecular Sequence Data’ and ‘Escherichia coli’. The benefit of our new graph-theoretical approach is that we can visualize how much these clusters have in common and how dependent they are. We can also identify clusters that consist of separate small clusters, which occur highly connected.

**Fig. 6:**
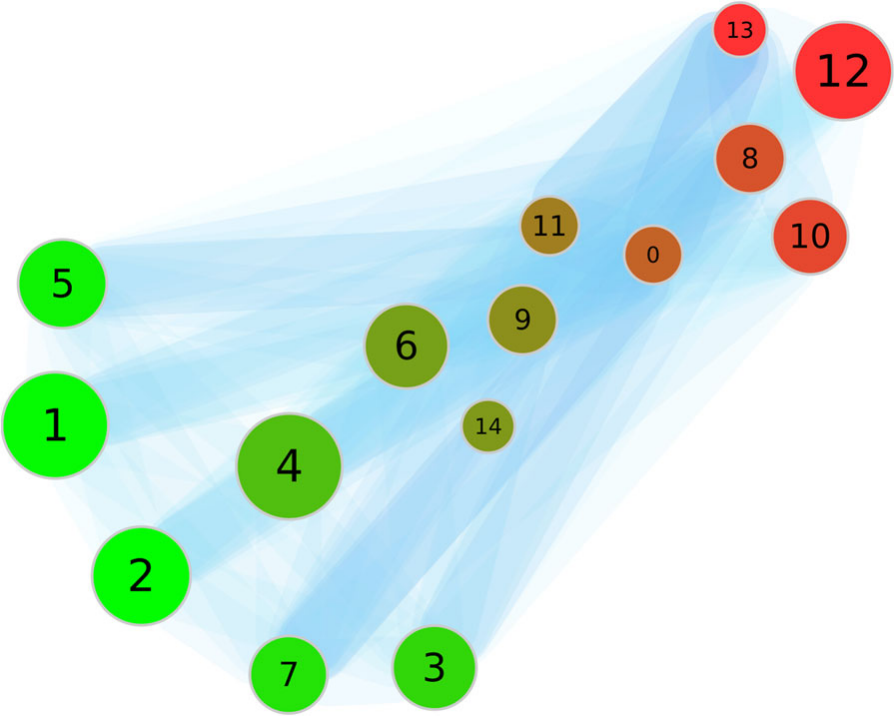
The partition of the first dataset with the distance *d*_2_. This picture shows the weighted graph of the clustering. The colors of the nodes indicate a high rate of documents from the respective queries (red: ’escherichia AND pili’; green ’cerevisiae AND cdc*’). The numbers identify the clusters. The size of a node scales with the number of documents inside. The edges and their width and color describe their weight. A blue edge has a greater weight

**Distance measure*d***_**2**_: The results of our clustering approach with distance *d*_2_ are shown in Fig. 6. The weighted graph of that clustering is now different. We got 14 clusters (Cluster 0 to 13) with clusters between 2 and 5 as well as 157 and 158 documents. We now have no isolated clusters.

In this clustering it is not easy to evaluate the different topics given through the search query by evaluating the edges within the weighted graph of the clustering. Thus we have colored the graph according to the rate of documents from each query. We would expect “clean” clusters, which means the clusters should have a high fraction of documents from only one query. We see several relatively clean clusters, for example 1 or 5, 2, 7 and 3. But those are not highly connected. The documents in between are mostly related to clusters which are not clearly assigned to one of both search queries. Thus, we could not clearly reproduce the results from [15] with this distance measure.

**Fig. 7:**
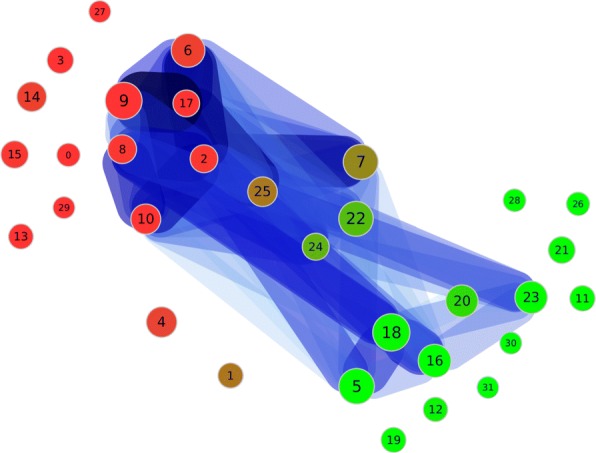
The partition of the first dataset with the distance *d*_3_. This picture shows the weighted graph of the clustering. The color of the nodes indicate a high rate of documents from the respective queries (red: ’escherichia AND pili’; green ’cerevisiae AND cdc*’). The numbers identify the clusters. The size of a node scales with the number of documents inside. The edges and their width and color describe their weight. A blue edge has a greater weight

**Distance measure*d***_**3**_: The results of our clustering approach with distance *d*_3_ are shown in Fig. 7. We now have one strongly connected set of clusters. It is no longer possible to separate any of the topic clusters induced by the search query. Thus, again we have colored the graph according to the fraction of documents from each query. We would expect “pure” clusters, which means the clusters should have a high fraction of documents from only one query. We get more pure clusters than with *d*_1_ and *d*_2_ but they are small. Most of the purest clusters are isolated and do not share documents with other clusters. Thus the result observed with *d*_2_ gets clearer. Only those clusters which cannot be clearly assigned to one of the search queries have edges within the weighted graph of the clustering.

Since all MeSH terms are weighted equally, those terms which are not significant but shared by many of documents, are scored higher, for example ‘Animals’ or ‘Microscopy’. And as a result, most documents have these terms in common. This explains the high connectivity of the resulting graph. Thus we could again not clearly reproduce the results from [15] with this distance measure.

## Discussion

We have shown a novel approach for document clustering considering hard clustering as well as soft clustering. We defined pseudostable sets and used the minMPS’-a approach to perform document clustering on a real-world example. We have introduced a integer linear programming and a greedy approach that gave valuable output on random instances as well as real-world data. This paper underlines that pseudostable sets have a broad application and can also be used to generalize other problems like document clustering. Since the problem is $\mathcal {NP}$-complete, we could only produce and evaluate approximate solutions.

The most important point to discuss is the impact of the proposed reformulation of soft clustering as a graph-theoretical problem. Doing so, we have a general problem formulation of soft clustering which offers an objective measure for other methods. Other things having an impact on the results – like similarity measures – can be identified as secondary, they do not provide an objective evidence of the clustering process. In addition, we discussed some points that make the problem $\mathcal {NP}$-complete.

More research in the future needs to be done on the special graph-theoretical background of our method. Since stable sets, cliques and pseudostable sets are under research and yet just partially well studied approaches, there is the need to bring optimization approaches from discrete mathematics to this application. However, we can now utilize the complete toolbox of graph theory, combinatoric optimization and discrete mathematics to our problem. Doing so, we hope to find better and faster heuristics, get optimal local solutions and improve the world of information retrieval.

As another, more general result, we can see that further research has to be done on evaluating the error given by the heuristics. Is it possible to find restrictions on *G* and *B* so that a solution in polynomial time is possible?

Because large graphs also increase the processing complexity, we identify the handling of such big data as an additional challenge. In the same course, it might be a good idea to focus also on novel strategies to implement an online algorithm version of the greedy approach, which could significantly improve the scalability.

We compared three simple similarity measures using textual data given by the abstract as well as keywords. We have shown that the clustering process itself is only valuable when choosing the right similarity measure. Although we have proven that the hard clustering and soft clustering approach using pseudostable or stable sets is valid, we might need to evaluate more similarity measures. Thus further research has to be done on similarity measures. We are planning to improve document management with this novel clustering approach and do more empirical evaluation by using test sets.

## Conclusions

The presented integer linear programming as well as the greedy approach for this $\mathcal {NP}$-complete problem lead to valuable results on random instances and some real-world data for different similarity measures. We could show that PS-Document Clustering is a remarkable approach to document clustering and opens the complete toolbox of graph theory to this field.

## Additional file

Additional file 1 **List of PMIDs used for analyses**. This is a simple text file containing the PMIDs, separated by newline characters. (CVS 13 kb)
